# *Eobowenia* gen. nov. from the Early Cretaceous of Patagonia: indication for an early divergence of *Bowenia*?

**DOI:** 10.1186/s12862-017-0943-x

**Published:** 2017-04-07

**Authors:** Mario Coiro, Christian Pott

**Affiliations:** 1 Department of Systematic and Evolutionary Botany, University of Zurich, Zollikerstrasse, 107 8008 Zurich, Switzerland; 2 Department of Palaeobiology, Swedish Museum of Natural History, Box 50007, SE-104 05 Stockholm, Sweden

**Keywords:** Cycadales, stomata, cuticle, biogeography

## Abstract

**Background:**

Even if they are considered the quintessential “living fossils”, the fossil record of the extant genera of the Cycadales is quite poor, and only extends as far back as the Cenozoic. This lack of data represents a huge hindrance for the reconstruction of the recent history of this important group. Among extant genera, *Bowenia* (or cuticles resembling those of extant *Bowenia*) has been recorded in sediments from the Late Cretaceous and the Eocene of Australia, but its phylogenetic placement and the inference from molecular dating still imply a long ghost lineage for this genus.

**Results:**

We re-examine the fossil foliage *Almargemia incrassata* from the Lower Cretaceous Anfiteatro de Ticó Formation in Patagonia, Argentina, in the light of a comparative cuticular analysis of extant Zamiaceae*.* We identify important differences with the other member of the genus, viz. *A. dentata,* and bring to light some interesting characters shared exclusively between *A. incrassata* and extant *Bowenia*. We interpret our results to necessitate the erection of the new genus *Eobowenia* to accommodate the fossil leaf earlier assigned as *Almargemia incrassata*. We then perfom phylogenetic analyses, including the first combined morphological and molecular analysis of the Cycadales, that indicate that the newly erected genus could be related to extant *Bowenia*.

**Conclusion:**

*Eobowenia incrassata* could represent an important clue for the understanding of evolution and biogeography of the extant genus *Bowenia,* as the presence of *Eobowenia* in Patagonia is yet another piece of the biogeographic puzzle that links southern South America with Australasia.

**Electronic supplementary material:**

The online version of this article (doi:10.1186/s12862-017-0943-x) contains supplementary material, which is available to authorized users.

## Background

The Cycadales have been regarded for their phylogenetic position and their number of plesiomorphic characters as the only group of pteridosperms that survived up to the present [1, 2]. They consequently play a crucial role in our understanding of the evolution of seed plants [1, 3]. The extant diversity of the cycads comprises ten genera and 346 species [4], traditionally distributed in the three families Cycadaceae, Stangeriaceae and Zamiaceae [5, 6]. However, more recent evidence based on molecular data tends to identify two main lineages, i.e. Cycadaceae and Zamiaceae, with the members of the Stangeriaceae sensu Stevenson [6] nested within Zamiaceae [7].

The Cycadales have long been considered to be a group with a rich fossil history, reaching its peak in diversity during the Mesozoic and declining up to the present [8], resulting in the Mesozoic being erroneously called the “Age of the Cycads”. In fact, cycads are commonly considered a member of the informal entity called “cycadophytes” that constitute several plant groups (Cycadales, Bennettitales, Nilssoniales) whose members resemble each other but are not closely related [9]. In contrast to the common perception, the dominant plant groups in mid-Mesozoic floras were in fact the Bennettitales and Nilssoniales (e.g. [9–15]), while Cycadales constituted only a minor portion of the vegetation. In addition, the results of recent molecular dating seem to indicate that most of the extant species diversity in cycads originated during the Late Miocene and Pliocene [16–18], and thus well after the Mesozoic.

Different hypotheses have been advanced to explain the apparently recent origin of the extant species of cycads. Some authors have interpreted these recent speciation events as a radiation triggered by increased aridification [17] or as the rebound after a mass extinction caused by the inception of an icehouse earth [16]. The resolution of this conundrum is hindered by our insufficient understanding of the relationships between fossil and extant diversity in cycads, which would allow us to independently test the trajectories of diversity through time [19], to validate the dates retrieved by the molecular analyses [20], and to fully understand the impact of climatic changes and tectonic events on the diversity of the group.

The fossil record of the ten extant genera of the Cycadales is indeed limited to a few Tertiary occurrences. Records considered to be reliable here include leaves and cuticle fragments assigned to *Cycas* from the Eocene of China [21], *Macrozamia* from the Oligocene of Australia [22], *Lepidozamia* from the Eocene of Australia ([23, 24], but see [25]), and *Ceratozamia* from the Oligocene–Miocene of Central Europe [26–28].

One of the best represented genera in the Tertiary record is *Bowenia* with two fossil species described from the Eocene of Australia [29] and Tasmania [30] as well as cuticular fragments with *Bowenia-*like morphology identified in the Eocene of Tasmania [31] and the Late Cretaceous of Central Australia [32]. A few other fossils are awaiting to be formally described [33]. *Bowenia* presents an interesting combination of characters that are uncommon in the other genera of the Cycadales (i.e. bipinnate leaves, stomata with non-sunken guard cells, a circularly arranged vascular bundle in the rachis; [6, 34, 35]). For this reason, the systematic classification and the phylogenetic placement of *Bowenia* are currently under debate. Some authors have identified *Bowenia* as a separate lineage in the Zamiaceae [5], others as the only member of a separate family (i.e. Boweniaceae; [35]) or as a close relative of *Stangeria* in the Stangeriaceae [6]. More recently, studies using molecular data [7] have tried to resolve the relationships between *Bowenia* and the rest of the cycads, with the placement of *Bowenia* as a close relative of *Stangeria* almost invariably rejected [7]. Instead, its placement as sister to the Ceratozamieae [17], Encephalarteae [16] or a clade comprising Ceratozamieae and Encephalarteae [7] is currently debated. In either case, *Bowenia* appears to be somewhat isolated from the other genera of the Cycadales, being separated by a relatively long branch from all other major clades [7, 17]. Despite the relatively good fossil record of *Bowenia*, the date of divergence from its sister group inferred from molecular data may imply a potentially long ghost lineage [16]. The phylogenetic isolation of *Bowenia* combined with its endemic distribution in Australia also complicates the resolution of its biogeographical history, with different methods yielding varying reconstructions [7].

Among Mesozoic taxa, only a few have been tentatively linked to extant groups. Some of the most interesting fossils come from the Lower Cretaceous Baquero Group in Patagonia, Argentina, which also yielded one of the highest diversities in cycad leaf taxa [36]. Among these are the leaf taxa *Mesodescolea* [37] and *Restrepophyllum* [38], which have been provisionally linked with extant *Stangeria* and *Zamia* (including *Chigua*)*,* respectively. Other cycadalean taxa from the Baquero Group, such as *Pseudoctenis ornata* A.Archang., R.Andreis, S.Archang. et A.Artabe [39], present interesting morphological similarities with members of extant Cycadales, but their relationships with any extant genus are controversial [40, 41].

In this contribution, we report our analyses of the leaf fossil *Almargemia incrassata* S.Archang. from the Anfiteatro de Ticó Formation of the Baquero Group, undertaken in the context of ongoing comparative studies of the cycadalean epidermis. Our results revealed that the fossils are different from the type of *Almargemia*, necessitating us to erect the new genus *Eobowenia* to accommodate leaves that share some important characters with extant *Bowenia*. We then test the placement of *Eobowenia* in the phylogeny of the Cycadales using an updated morphological matrix in combination with molecular data. Based on the results of the phylogenetic analyses, we discuss the implications of *Eobowenia* for the biogeographical history of *Bowenia.*

## Methods

### Specimens investigated

The fossil specimens examined for this study were first described by Florin [42] and later by Archangelsky [43]. The specimens examined for *Almargemia incrassata* are stored in the Natural History Museum (NHM), London, UK, to which they were donated as duplicates by Sergio Archangelsky in 1960, under accession numbers v52264 (macrofossil) and v52265 (cuticle slide). The specimens examined for *A. dentata* Florin are stored in the Swedish Museum of Natural History (NRM), Stockholm, Sweden, under accession numbers S085614–S085619. Two of the original specimens examined by Heer [44] and Florin [42] could be traced down at the Museu Geológico, Lisbon, Portugal (accession numbers 23,213 and 23,217). Sources of the samples from extant species are listed in Additional file 1: Table S1. The slides produced from the latter are stored at the Department of Systematic and Evolutionary Botany of the University of Zurich, Zurich, Switzerland.

### Extant material preparation

Whole leaves were fixed in 50% Ethanol. Sections of a leaf of *Bowenia serrulata* (W.Bull) Chamb. were stained and mounted according to Coiro and Truernit [45]. Cuticles were isolated by immersing leaf fragments in a 2:1 mixture of 30% hydrogen peroxide and 85% ethanol, warmed up to 60 °C until the leaf fragments turned transparent. Cuticles were then rinsed in distilled water and cleaned using a fine brush. Cuticles of *Macrozamia plurinervia* (L.Johnson) D.L.Jones were isolated using overnight maceration in 10% Cr_2_O_3_. Cuticles were then stained in Auramine O (Sigma; 0.01% *w*/*v* in 0.05 M Tris/HCl, pH 7.2) for 10–15 min (see [46]). They were then rinsed with water and mounted in glycerol. All extant samples used for our comparative analyses are listed in Additional file 1: Table S1.

### Microscopy and image analysis

For light and epifluorescence microscopy, slides with fossil cuticles were observed using a Nikon Eclipse LV100ND microscope (*Almargemia incrassata*) or an Olympus BX-51 light microscope, which was modified for epifluorescence microscopy, and photographed with an Olympus DP-71 digital camera (*A. dentata*). Cuticles of extant cycads and whole-mount leaf samples were observed using a Zeiss Axioscope fitted with a Zeiss 38 HE fluorescence filter.

Confocal observations of fossil samples were made using a Nikon A1-Si laser-scanning confocal microscope, with two excitation lines: 488-nm line of 50-mW sapphire laser and 561-nm line of 50-mW sapphire laser (Coherent Inc., Santa Clara, California, USA). The autofluorescence signal was collected with two different photomultiplier detectors with the following wavelength emission windows: 500–550 nm for the 488-nm laser, 570–620 nm for the 561-nm laser. PS-PI stained samples and Auramine O stained cuticles of *Bowenia spectabilis* were observed using a Leica TCS SP8 microscope. Excitation was obtained using a 488 nm laser for the PI and a 405 nm diode laser for the Auramine O. Raw images were analysed and measured using the software FiJi [47]. Brightness and contrast were adjusted using the “auto” option in the software. Confocal stacks were combined using a Maximum Intensity projection. Scans of freshly cut leaves of extant cycads were taken at 1200 dpi using an Epson Perfection V600 Photo J252A scanner.

### Phylogenetic analyses

To test the placement of *Eobowenia* in the phylogeny of the Cycadales, we coded *Eobowenia* and *Almargemia* by incorporating the new data from our investigation in a modified version of the morphological matrix from Martinez et al. [48]. We removed the taxa that had no character overlap with our foliage taxa from the matrix. We then changed some of the character states in the light of our comparative data. In detail, we coded *Bowenia* and *Stangeria* as having hypostomatic leaflets (character 49) and oblong stomata (character 51), *Stangeria* as having one accessory cell layer (character 53), *Bowenia* as having longitudinally-oriented stomata (character 52) and Bennettitales as having both flush and sunken stomata (character 50). We also added a character for the substomatal complex thickenings, coded as present in *Eobowenia, Encephalartos, Macrozamia* and *Bowenia,* and absent in the other extant Cycadales except *Lepidozamia*. The state for such character in other fossil cycads as well as in the outgroups was coded as unknown. *Eobowenia* was coded conservatively regarding the architecture of the leaf, with leaf dissection coded as pinnate (character 24) and midrib coded as absent (character 32).

Two different sets of analyses were performed: first, we analyzed the morphological matrix separately, and secondly we combined the morphological matrix with the molecular matrix from Salas-Leiva et al. [7]. All analyses were conducted using both Maximum Parsimony (MP) as implemented in PAUP* ver 4.0b10 [49] and Bayesian Inference (BI) as implemented in MrBayes ver 3.2.6 [50]. Search for the MP trees was performed using heuristic search with 1000 addition replicates and random addition sequence of taxa, and the bootstrap analysis was conducted for 1000 replicates using 10 searches per replicate and keeping only one tree per replicate. We also ran an analysis with the relationships between the extant cycad genera from Salas-Leiva et al. [7] forced as a backbone constraint. The BI analyses were executed using two runs of four chains (one cold and three hot chains), with 1 million generations for the morphology-only analysis and 5 million generations for the combined morphological-molecular analysis. In the morphology-only analyses, we used the mk_inf_ model [51] with gamma-distributed rate variation. In the morphological-molecular analyses, we set one partition for each of the markers plus one morphological partition. For all molecular markers we used a GTR plus gamma model, and for the morphological partition we used a mk_inf_ plus gamma model. After discarding 25% of the trees as burn-in, trees were summarized as consensus trees including all compatible splits. Characters were then reconstructed on the trees using Maximum Parsimony as implement in Mesquite ver 3.03 [52] to identify synapomorphies.

We also used the modified morphological matrix to test the different placements of *Eobowenia* in relation to the extant genera of the Cycadales using Mesquite ver 3.03 [52]. We edited a tree of the extant genera according to the topology of Salas-Leiva et al. [7], and counted the length of the trees obtained by moving the placement of *Eobowenia* by hand.

## Results

### Systematic Palaeontology

*Order* – Cycadales Pers. ex Bercht. et J.Presl.

*Family –* Zamiaceae Miq.

*Subfamily –* Bowenioideae Pilg. in H.G.A.Engler et K.A.E.Prantl.

*Genus – Eobowenia* M.Coiro et C.Pott gen. nov.

*Type: Eobowenia incrassata* (S.Archang.) M.Coiro et C.Pott comb. nov., from the Aptian (Lower Cretaceous) of Patagonia, Argentina.

*Diagnosis:* Emended from Archangelsky [43]. Leaves pinnate. Midrib delicate. Leaflets subopposite, insertion more acute towards the apex, oblong. Leaflet base broad. Leaflets with serrate margin. Veins parallel to the margin, converging at the base of the leaflet. Leaflets hypostomatic. Stomata with guard cell poles raised with respect to the aperture. Guard cells at same level with the epidermis, arranged longitudinally with respect to the leaflet axis. Stomatal complex monocyclic. Subsidiary cells with differentiated cuticle. Polar subsidiary cells sometimes differentiated from the lateral ones. Substomatal cells with thickened secondary cell walls. Epidermal cells elongated parallel to the leaflet axis, with darker-staining short cell distributed in rows of short cells. Anticlinal cell walls slightly wavy or concave.

*Etymology*: From Greek Ἕως, dawn, and the name of the extant cycad *Bowenia*.

**Fig. 1:**
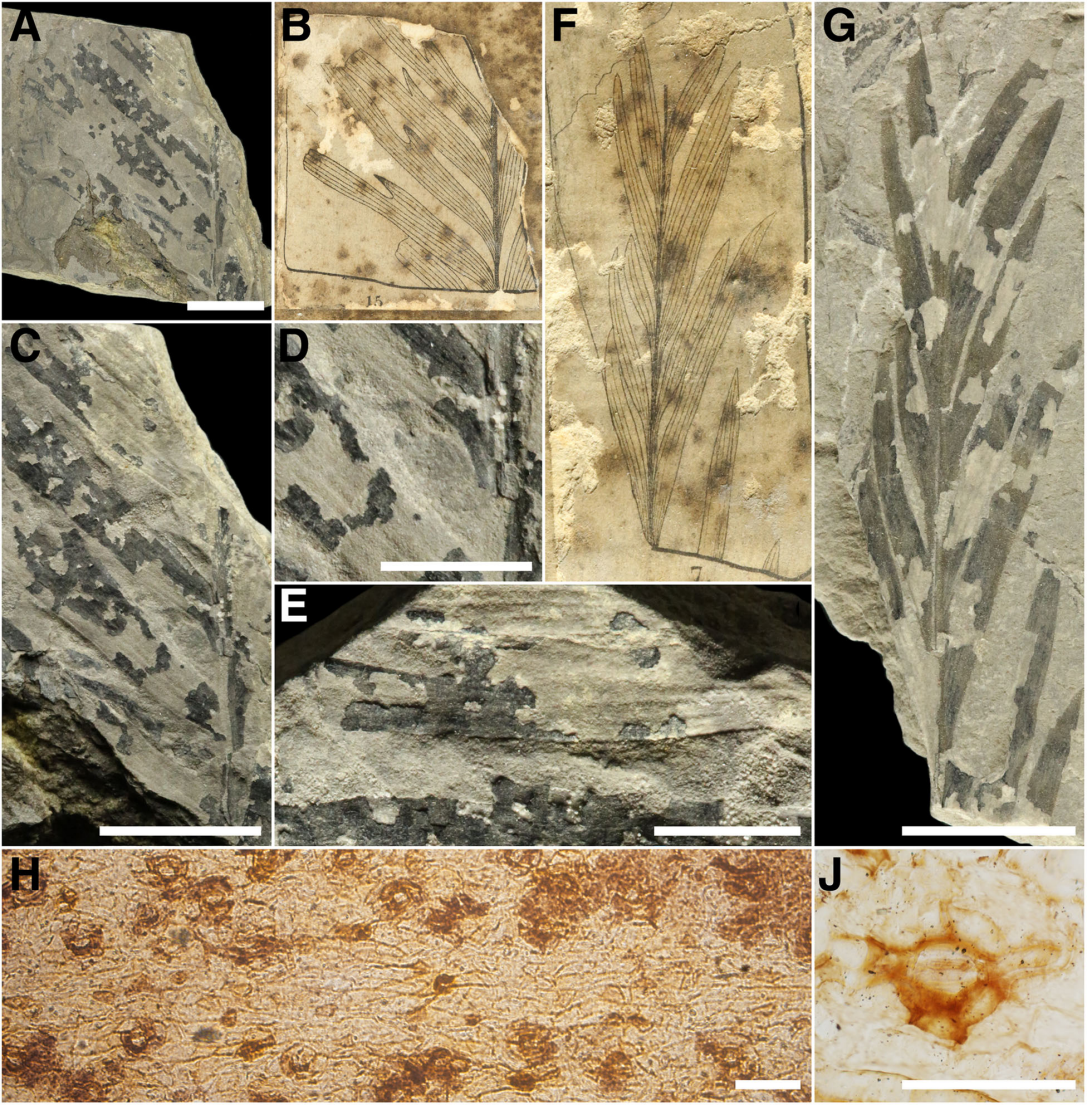
*Almargemia dentata* from the Lower Cretaceous of Portugal. **a**-**e** Several leaflets of the middle portion of a leaf, note the lobe-like teeth on the basiscopic margin of the leaflets, specimen 23,217; **f**-**g** More apical portion of a leaf where leaflets are inserted in much lower angles, specimen 23,213, both specimens stored in the Museu Geológico, Lisbon, Portugal; **h** Overview of lower epidermis, note the intercostal fields separated by a costal field (horizontal, middle of image), specimen S085620; **j** Close-up of a stoma, specimen S085620, stored in the Swedish Museum of Natural History, Stockholm, Sweden. Scale bars: **a**, **c**, **g** 1 cm; **d**, **e** 5 mm; **h** 100 μm; **j** 25 μm

*Remarks*: Based on the new characters identified and a re-evaluation of the epidermal anatomy of extant *Bowenia* and fossil *Almargemia dentata* (Fig. 1) and *A. incrassata,* we erect the new genus *Eobowenia.* In our opinion, the leaflets of *A. incrassata* share interesting characters with *Bowenia*, but are distinct enough to deserve the institution of a new genus. *Eobowenia* is distinguished from *Almargemia* by the leaflets with serrate margin, the veins converging at the base of the leaflets, the guard cells at the same level of the epidermis arranged longitudinally with respect to the leaflet axis, and the monocyclic stomatal complexes. From *Bowenia*, it is distinguished by the presence of darker-staining cells arranged in rows, the broad attachment of the leaflets, and the smaller size of the leaflets.

*Eobowenia incrassata* (S.Archang.) M.Coiro et C.Pott comb. nov.

1966 *Almargemia incrassata* – Archangelsky, p. 267; pl. I, Figs. 3, 4; pl. III, Figs. 13, 14; Text-Figs. 6–10, 13.

*Diagnosis:* As for the genus, with the following additions: Leaflet base with constricted acroscopic margin and decurrent basiscopic margin. Striations are visible in between the veins.

*Holotype:* LP6255, Museo de Ciencias Naturales, La Plata, Argentina.

*Remark on types:* Specimen LP6255*,* published by Archangelsky [43], automatically becomes the holotype of the new combination and the new genus*.* However, we chose specimen v52265 (a cuticle slide obtained from LP6255) as epitype; it serves as interpretative type because it perfectly presents the combination of characters necessitating the erection of the new genus.

*Type locality:* Estancia Bajo Grande, Santa Cruz Province, Argentina (not Bajo Tigre as erroneously reported by Archangeslky [43], see [53]).

*Type unit and age*: Baquero Group, Anfiteatro de Ticó Formation, *Auracarites* Bed. Lower Cretaceous (Aptian).

**Fig. 2:**
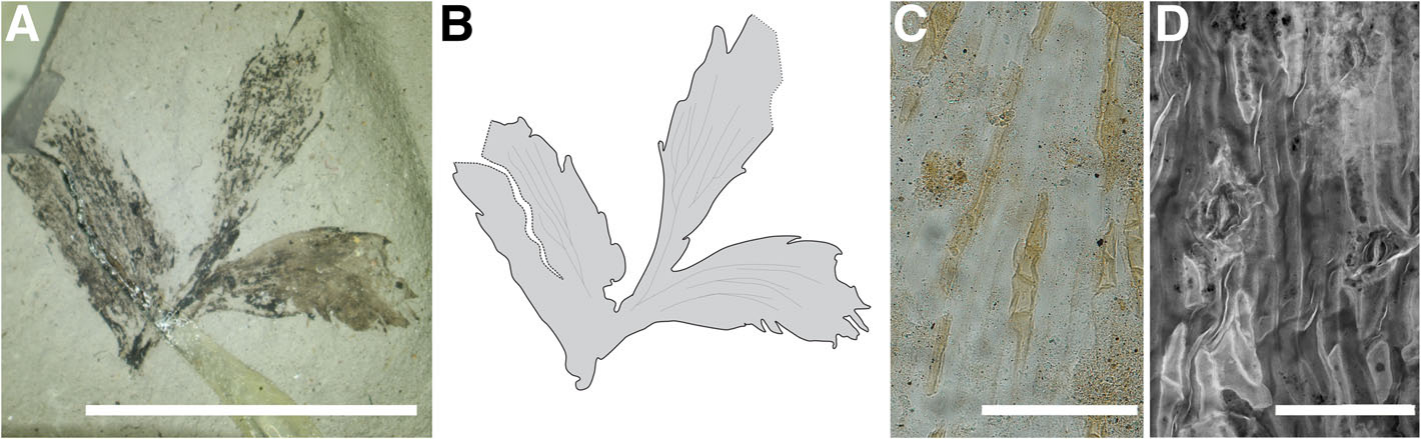
*Eobowenia incrassata* gen. nov., comb. nov., from the Aptian (Lower Cretaceous) of Patagonia, Argentina. **a** Overview of an apical leaf fragment (specimen v52264). **b** Interpretative drawing of the specimen, showing potentially dichotomizing veins. **c** Light microscopy image of the adaxial cuticle, note the short rows of more heavily cutinised epidermal cells (specimen v52265). **d** CLSM image of the abaxial cuticle, note the darker staining epidermal cells (specimen v52265). Scale bars: **a** 10 mm, **b** 1 mm, **c** 100 μm

*Description: Eobowenia incrassata* (Fig. 2) is represented by two fragmentary specimens [43]. The three (probably terminal) leaflets on specimen v52264 clearly show the serrate margin, the attachment of the leaflets, and the fine striations present between the veins on the leaflets (Fig. 2 a). These characters were already identified as diagnostic for the species by Archangelsky [43]. The preserved portions of the leaflets are 7.4–9.3 mm long and up to 3.5 mm wide.

**Fig. 3:**
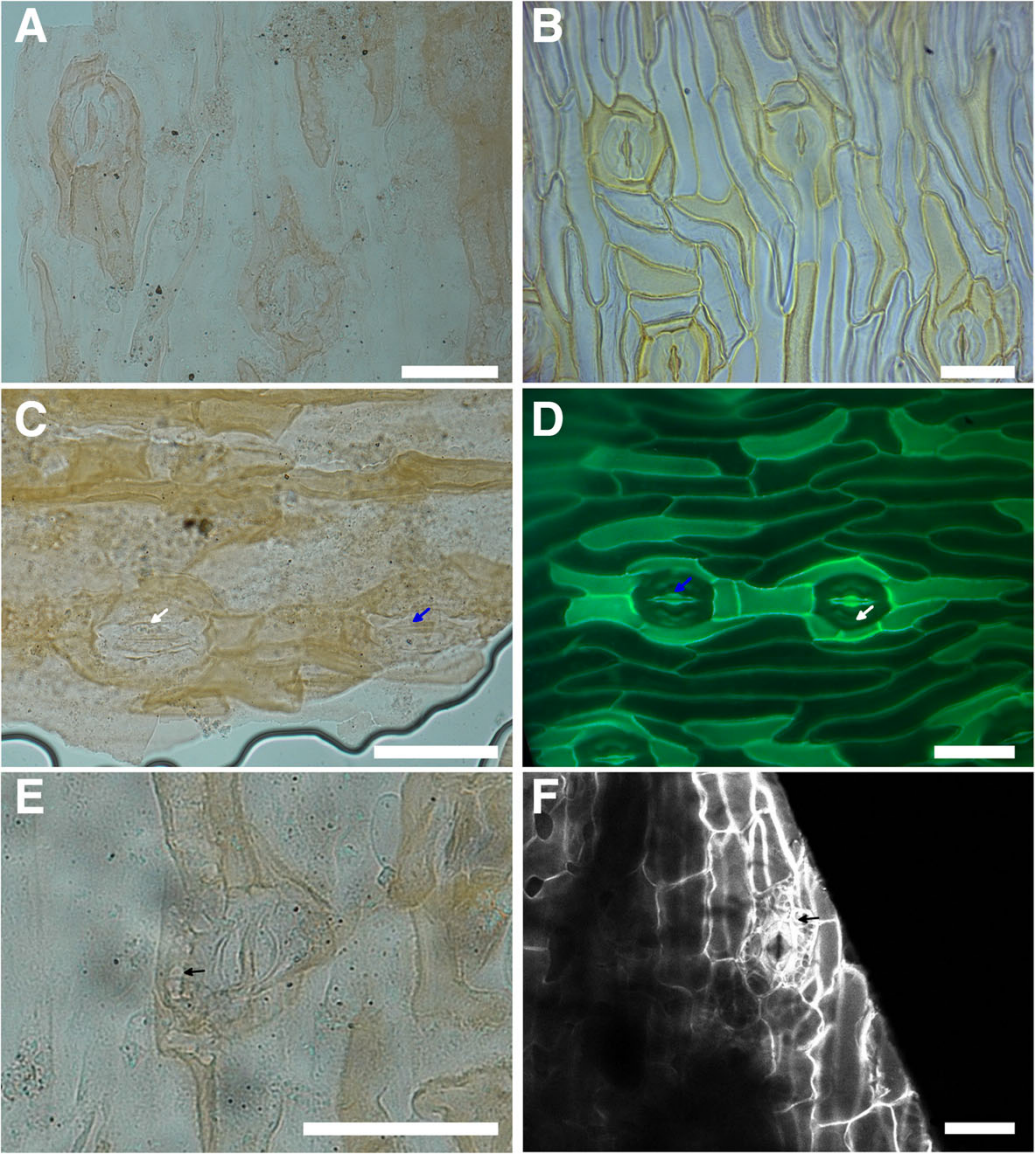
Comparison between the cuticle of *Eobowenia incrassata* (**a**, **c**, **e**, specimen v52265) and *Bowenia spectabilis* (**b**, **d**, **f**). **a** Stomata on the abaxial cuticle of *Eobowenia incrassata,* showing the monocyclic architecture and the darker-staining pavement cells. The thickenings of the substomatal complexes are preserved under some stomata. **b** Stomata on the abaxial cuticle of *Bowenia spectabilis,* showing similar monocyclic stomatal architecture and the darker-staining (thickly cutinised) pavement cells. **c** Detail of two stomatal complexes in *Eobowenia incrassata*. The distal thickening (blue arrow) and the marginal ridge (white arrow) are clearly shown. **d** Detail of the stomatal complex in *Bowenia spectabilis.* Distal thickening (blue arrow) and the marginal ridge (white arrow) are present in the cuticle of the guard cells. **e** Detail of a stomatal complex in *Eobowenia incrassata* showing the partially preserved substomatal complex with secondary thickenings (black arrow). **f** The substomatal complex shown in a confocal stack of PI-stained leaflets of *Bowenia spectabilis.* Thickenings are indicated by the black arrow. **b** and **d** are light micrographs (**b**) or fluorescence pictures (**d**) of the cleared cuticle of *Bowenia spectabilis*. Scale bars: 50 μm

**Fig. 4:**
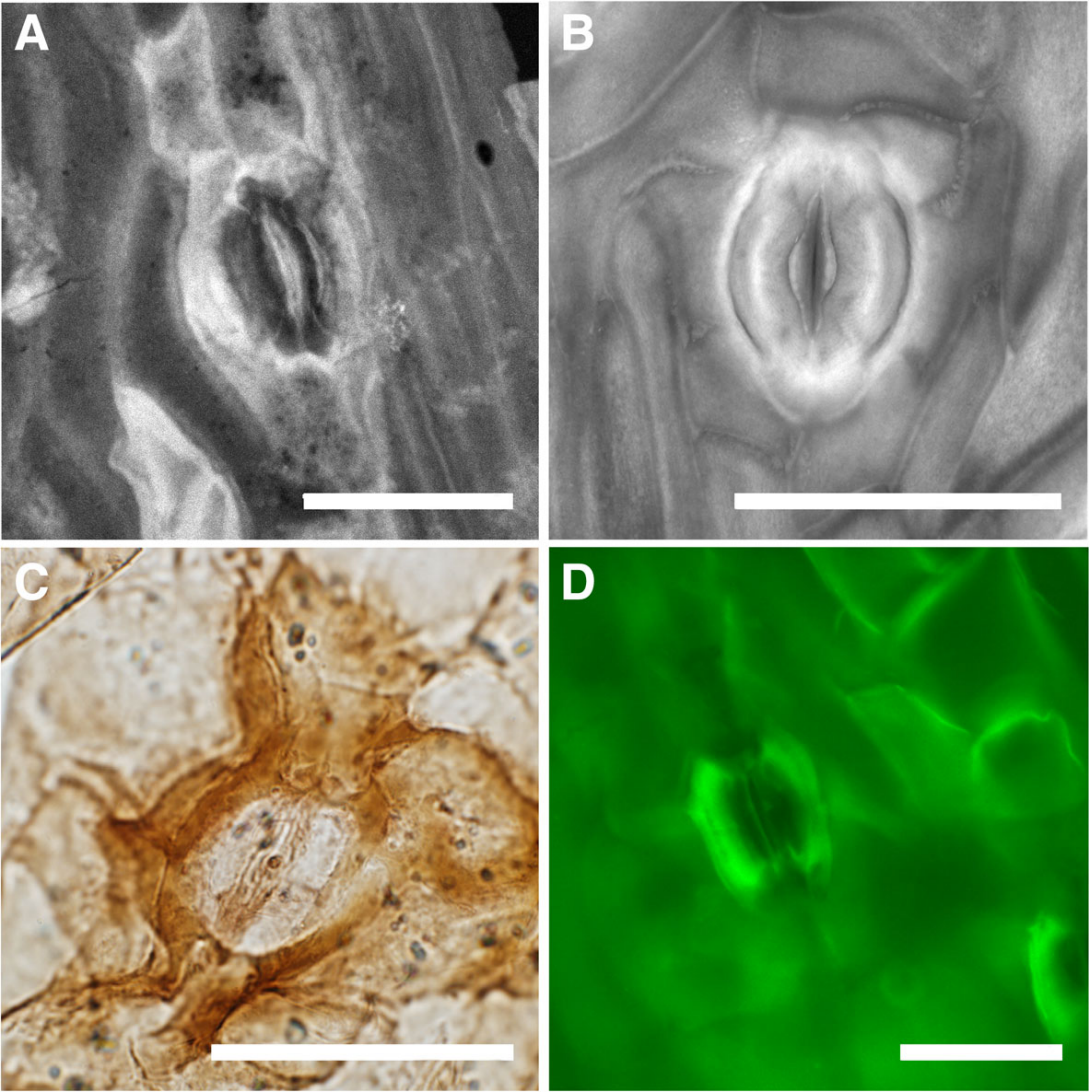
Comparison between the stomatal complexes of *Eobowenia incrassata* (**a**, specimen v52265), *Bowenia spectabilis* (**b**), *Almargemia dentata* (**c**, specimen S085614) and *Macrozamia plurinervia* (**d**). **a** Stomatal complex in *Eobowenia incrassata* with flush guard cells, thickening of the apertural cuticle of the guard cells and cuticular ridge. **b** Stomatal complex in *Bowenia spectabilis,* showing similarities to *Eobowenia.* **c** Stomatal complex in *Almargemia dentata,* showing the sunken guard cells. **d** Stomatal complex in *Macrozamia heteromera,* showing similarly sunken guard cells. **a** and **b** are maximum intensity projections of confocal stacks, **c** a light micrograph and **d** a fluorescence micrograph. Scale bars: 50 μm

The cuticle fragments examined show that the leaflets are hypostomatic with epidermal pavement cells longitudinally elongated parallel to the leaflet axis (Fig. 2 c, d). Ordinary epidermal cells are elongate and moderately cutinised. On the adaxial side, rows of cells with thicker cuticle than the ordinary pavement cells can be observed (darker staining; equivalent to the thin-walled cells of most Zamiaceae (see [54])), which seem to be arranged preferably in rows of short cells. The anticlinal walls of these dark-staining cells tend to be slightly concave. On the abaxial side, rows of darker staining cells as well as single darker staining cells are present. The stomata are confined to the abaxial side and are distributed uniformly in broad intercostal bands on the leaflet surface, with the guard cells oriented longitudinally (Fig. 3a, c). Guard cells are in average 38.63 (35.53–42.30) μm long and 17.90 (16.86–19.71) μm wide, with an aperture that is 20.89 (14.78–23.98) μm long. The stomatal complexes are monocyclic, with four to six subsidiary cells that have a thicker, darker staining cuticle than the ordinary pavement cells. The cuticle of the guard cells presents a ventral thickening in the correspondence of the aperture as well as ridges that run parallel to the dorsal wall (Fig. 2 d; Fig. 3 c, e; Fig. 4 a). In some stomatal complexes, is possible to observe differentially thickened or perforated cell walls, which are similar to the cell wall of the substomatal complex in extant *Bowenia* (Fig. 2 d; Fig. 3 e).

*Remarks:* The characters that separate the fossils assigned to *Almargemia incrassata* from those assigned to *A. dentata* and link the former with *Bowenia* are depicted in Table 1. The allocation of *A. incrassata* to the new genus *Eobowenia* retains *A. dentata* as the only representative of *Almargemia*.

**Table 1:** Summary of the characters distinguishing *Almargemia dentata, Eobowenia incrassata* and *Bowenia*

Species	Venation	Teeth	Guard cells	Stomatal apparatus	Stomatal orientation
*Almargemia dentata* (Heer) Florin	Parallel	Lobe-like	Sunken	Dicyclic	Random
*Eobowenia incrassata* (S.Archang.) M.Coiro et C.Pott	Convergent at the base	Simple, glandular?	Level with epidermis	Monocyclic	Longitudinal
*Bowenia serrulata* (W.Bull) Chamb.	Convergent at the base	Simple	Level with epidermis	Monocyclic	Longitudinal
*Bowenia spectabilis* Hook. ex Hook.f.	Convergent at the base	Absent-simple	Level with epidermis	Monocyclic	Longitudinal
*Bowenia papillosa* R.S.Hill	?	?	Level with epidermis	Monocyclic	Longitudinal
*Bowenia eocenica* R.S.Hill	Convergent at the base	Simple	Level with epidermis	Monocyclic	Longitudinal

### Phylogenetic analyses

The MP analysis of the modified morphological matrix of Martinez et al. [48] resulted in 242 equally parsimonious trees of 196 steps. In the strict consensus tree, *Bowenia* and *Eobowenia* are in a polytomy with most of the other fossil taxa. This is due to the uncertainty in the placement of *Stangeria* and *Mesodescolea*, which could be equally parsimoniously placed as sister to *Eobowenia* plus *Bowenia*, sister to *Bowenia* alone with *Eobowenia* as sister to this clade, or in a clade with other fossil taxa (*Kurtziana*, *Pseudoctenis*, *Sueria*, *Mesosingeria*). In the bootstrap analysis, a sister group including *Eobowenia* and *Bowenia* does not receive support, being retrieved in only 50% of the bootstrap replicates. Forcing the molecular backbone constraint from Salas-Leiva et al. [7] on the modified morphological matrix of Martinez et al. [48] results in 594 trees of 222 steps. In the consensus tree, only a few relationships are resolved, but *Bowenia* and *Eobowenia* result sister taxa. In the Bayesian consensus tree of the morphology-only analysis, a clade including *Eobowenia* as sister to *Bowenia*, *Stangeria* and *Mesodescolea* receives a weak support (0.62 posterior probability).

**Fig. 5:**
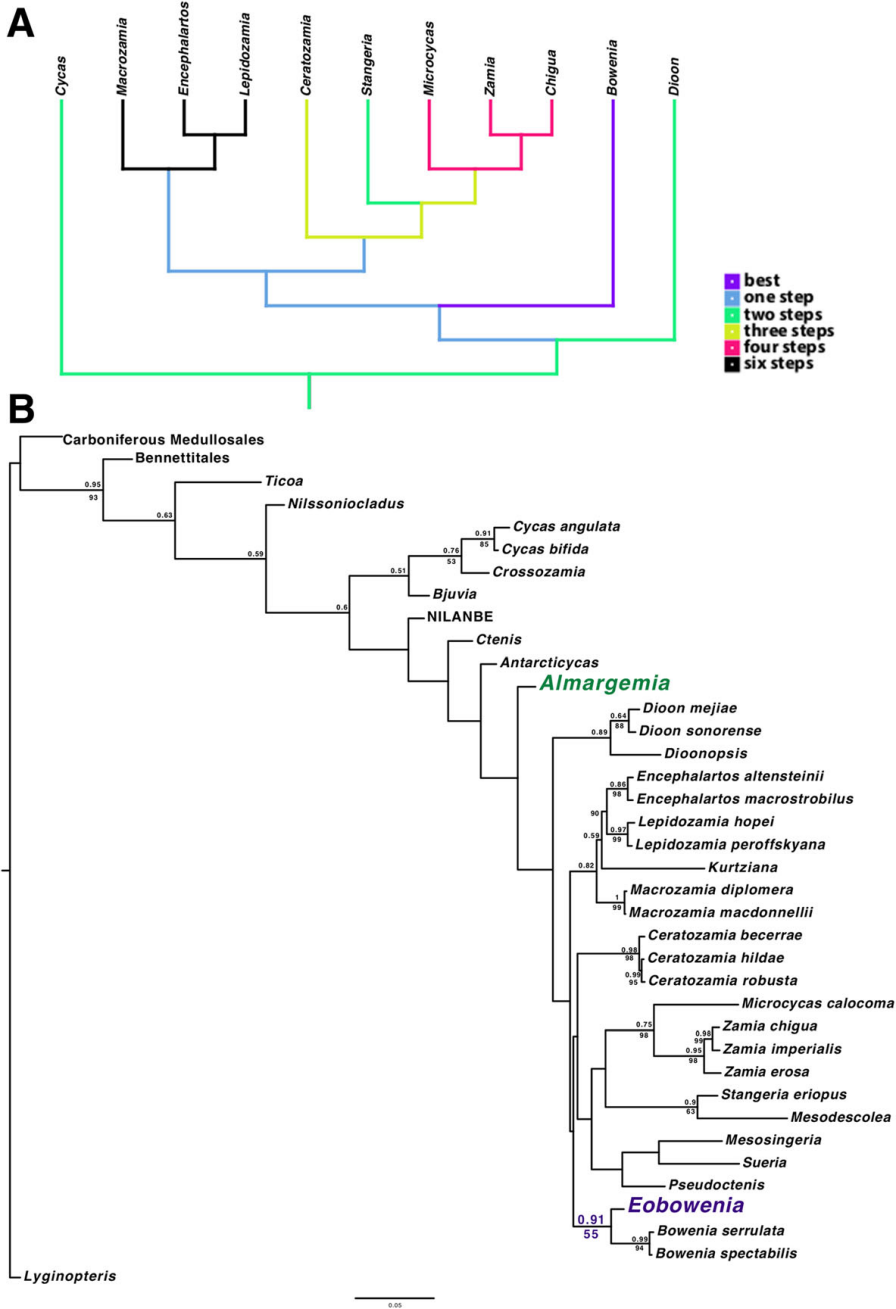
Phylogenetic placement of *Eobowenia*. **a** Number of steps necessary to place *Eobowenia* on a tree based on Salas-Leiva et al. [7] using the modified Martinez et al. [48] matrix. Placement as sister to *Bowenia* results in the shortest trees, but other placements are only marginally less parsimonious. **b** Consensus with all compatible split from the Bayesian analysis of the modified modified Martinez et al. [48] matrix combined with the Salas-Leiva et al. [7] molecular matrix. Posterior probability more than 0.5 are shown above the branches, and Maximum Parsimony bootstrap support over 50% is shown below the branches

The MP analysis of the combined matrix resulted in 368 trees of 2925 steps. The consensus tree is poorly resolved, but *Eobowenia* results sister group to the two *Bowenia* species. In the bootstrap analysis, this relationship is weakly supported (55% of the bootstrap replicates). The BI analysis of the combined morphological-molecular matrix strongly supports a placement of *Eobowenia* as sister of the two species of *Bowenia* (0.91 posterior probability) (Fig. 5 b). The presence of flush guard cells (char 50) and the absence of encircling cells (char 53) represent synapomorphies of the *Eobowenia* and *Bowenia* clade in this topology, whereas the presence of a thickened substomatal apparatus (char 89) is ambiguously resolved as either synapomorphic for *Ebowenia* and *Bowenia* or plesiomorphic for all Zamiaceae except *Dioon* (Additional file 2: Figure S1).

Using the topology from Salas-Leiva et al. [7] as a backbone and moving *Eobowenia* by hand, the shortest tree is obtained with *Eobowenia* as sister to *Bowenia* (131 steps). A placement of *Eobowenia* as sister to *Bowenia* plus Ceratozaminae and Encephalartinae, sister to Ceratozaminae plus Encephalartinae, or sister to either Encephalartinae or Ceratozaminae is one step longer. Placement as sister to *Dioon, Cycas,* Zamiaceae or *Stangeria* requires two more steps. Placement as sister to *Ceratozamia*, sister to *Stangeria* plus *Microcycas* plus *Zamia* plus *Chigua* or sister to *Microcycas* plus *Zamia* plus *Chigua* requires three more steps. Placement in any position in the *Microcycas-Zamia* clade requires four more steps, and placement in any positions in the *Macrozamia*-*Encephalartos*-*Lepidozamia* clade requires six more steps (Fig. 5 a).

## Discussion

Our reinvestigation of the original specimens of *Almargemia incrassata* and *A. dentata* revealed remarkable differences between the two species. These differences necessitated the transfer of *A. incrassata* to a different genus, viz. *Eobowenia* gen. nov.

### *Comparison of* Almargemia dentata *and* Eobowenia incrassata

The leaves from the Lower Cretaceous of Portugal later referred to by Florin [42] as *Almargemia dentata* were first described by Heer [44] as *Ctenidium dentatum* Heer and *C. integerrimum* Heer. In the generic diagnosis, Heer [44] distinguished *Ctenidium* from *Ptilophyllum* and *Ptilozamites* by the decurrent leaf bases and from *Ctenis* by the absence of vein anastomoses. Florin [42] investigated the epidermal anatomy of the specimens described by Heer [44] in detail and, as a consequence, transferred both species in the new combination *Almargemia dentata,* correctly recognizing that the genus name selected by Heer [44] was pre-occupied by a genus of extant mosses. The main diagnostic epidermal characters of *Almargemia* according to Florin [42] were the predominantly incompletely amphicyclic haplocheilic stomata, arranged irregularly in stomatal bands running between the veins on the abaxial surface of the leaflets, the sunken guard cells and the presence of both weakly and strongly cutinised pavement cells. Macromorphologically, the diagnostic characters included slightly contracted leaflet bases, parallel (rarely dichotomizing) venation and the presence of lobe-like teeth (Fig. 1 a, b, e).

When Archangelsky [43] described *Eobowenia incrassata* (as *Almargemia incrassata*)*,* he decided to assign such specimens to *Almargemia* on the base of the serrate margin of the leaves (erroneously identified as ‘dentate’ by Archangelsky [43]) and the differently thickened cutinization of the epidermal cells. However, most of the other diagnostic characters of *A. dentata* are absent in *E. incrassata* (Table 1). The stomatal characters are strikingly different (Fig. 4), with *E. incrassata* having guard cells at the same level of the epidermal cells, monocyclic stomatal complexes and longitudinally oriented guard cells, while *Almargemia dentata* has stomata sunken below the epidermal level, as in most extant Zamiaceae, incompletely amphicyclic stomatal complexes and randomly oriented guard cells. To use only the differentially thickened cutinisation of the epidermal cells is, in our opinion, too weak a character to assign the fossils in question (viz. *Eobowenia incrassata*) to *Almargemia,* because of their common presence in most members of extant Zamiaceae [34, 41, 54]. Moreover, the dentation of the margin in the two species is quite different, with *E. incrassata* having relatively small, acute teeth and *A. dentata* having larger, lobe-like teeth. For these reasons, we reconsider the allocation made by Archangelsky [43] by erecting a new genus because a new generic definition is needed for this fossil taxon.

### *Comparison of* Eobowenia incrassata *with other fossil cycadophytes*

The leaves of *Eobowenia incrassata* are easily distinguishable from all other cycadalean leaf taxa described from the Baquero Group (i.e. *Ticoa*, *Mesosingeria*, *Mesodescolea*, *Sueria*; [40, 43]) by their leaf shape and epidermal anatomy (see [40, 43]). Among other Mesozoic cycadophyte leaves with parallel venation, *E. incrassata* differs from *Pseudoctenis* [55] by its basally converging veins, the serrate margin and by epidermal characters (i.e. guard cells at the same level as the epidermal cells, darker-staining pavement cells, longitudinally elongated pavement cells), and from *Ctenis* [55] by the absence of vein anastomoses as well as the very different cuticle. It differs from segmented *Nilssonia* leaves [55] by the lateral attachment of the leaflets and the anatomy of the cuticle and from *Encephalartites* by the leaf base that is contracted only on the acroscopic side, and by the oblong leaflets. *Eobowenia incrassata* is distinguished from any segmented bennettitalean leaf by the haplocheilic architecture of the stomata in contrast to the syndetocheilic architecture characterising bennettitalean leaves [19, 14].

A similar combination of differentially thickened epidermal cells, monocyclic stomatal complexes and guard cells at the same level with the aperture is present in some species assigned to the tentative pteridosperm genus *Stenopteris*. Monocyclic stomatal complexes with differentiated subsidiaries are present in *S. nana* T.M.Harris from the Bajocian of Yorkshire [55], but the overall morphology of the leaf easily distinguishes this species from *Eobowenia incrassata*. Another interesting species is *S. cyclostoma* K.Saiki, T.Kimura et J.Horiuchi, from the Lower Cretaceous Choshi Group of Japan [56]. The cuticle of this species presents many similarities with *E. incrassata* including the rows of dark staining cells [56], but presents a very dissimilar morphology of the leaf. However, there are differences even at the cuticular level, with *S. cyclostoma* being clearly amphistomatic and having an external vestibulum. Moreover, we were not able to identify the peculiar perforations of the substomatal complexes in the illustrations of Saiki et al. [56]. The cycad-like characters of *S. cyclostoma* are definitely interesting, but a more thorough discussion would include a revision of the morphology of the entire genus, and falls outside the scope of the present investigation.

### *Comparison of* Eobowenia *and* Bowenia

Our reinvestigation pinpoints numerous similarities between *Eobowenia incrassata* and the extant cycad genus *Bowenia* (Table 1). Among the most interesting characters are the flush guard cells, which clearly separate *Eobowenia* from *Almargemia dentata* as well as from all Zamiaceae and Cycadaceae sensu Stevenson [6] (Fig 4, Additional file 3: Figure S2; Additional file 4: Figure S3; Additional file 5: Figure S4; Additional file 6: Figure S5; Additional file 7: Figure S6). The cuticle of the guard cells also presents cuticular thickenings both on the dorsal and ventral surfaces, and single cuticular ridges running parallel to the dorsal wall of the guard cells (Fig. 3 c, d; Fig. 4 a, b), the monocyclic stomatal complexes (Fig. 3 a, c), and the presence of substomatal cell complexes with secondarily thickened walls (Fig. 3 e). The first set of characters is present among extant cycads in *Bowenia* and *Stangeria*, with some differences between the two genera [34]. Monocyclic stomatal complexes with stomata at the same level with the epidermis are restricted in extant Zamiaceae to *Bowenia* [41, 57, 58] (Additional file 6: Fig. S4). The perforations associated with some of the stomatal complexes in *Eobowenia incrassata* presents some striking similarities to the substomatal complex in *Bowenia,* which present secondarily thickened cell walls. This structure was interpreted by Greguss [34] as a perforation of the subsidiary cells, not dissimilar to the condition present in *Cycas* [54], where all epidermal cells present perforations of the inner periclinal wall. The structures in *Eobowenia incrassata* more closely resemble the structures in *Bowenia* (which also occur but are less developed in some species of *Encephalartos* and *Macrozamia*; see Additional file 7: Figure S6) in being mostly restricted to the substomatal complexes (Fig. 3 e, f). The main difference between the epidermis/cuticles of *Eobowenia incrassata* and *Bowenia* is the presence of files of short cells with thickened cuticle in the former. This character has been compared to the state present in *Ceratozamia* [57] by Kvaček [28]*,* where files of short, dark-staining cells are present on both surfaces of the leaflets. However, the slightly concave and sometimes wavy anticlinal cell walls of the dark-staining cells is closer to the cuticle of the cell files present in *Dioon* (Additional file 3: Figure S2 C, D) [41]. Darker staining cells are present in *Bowenia,* but they are organised as single or small groups of cells, commonly of the same length as the other epidermal cells (Fig. 3 b, d).

**Fig. 6:**
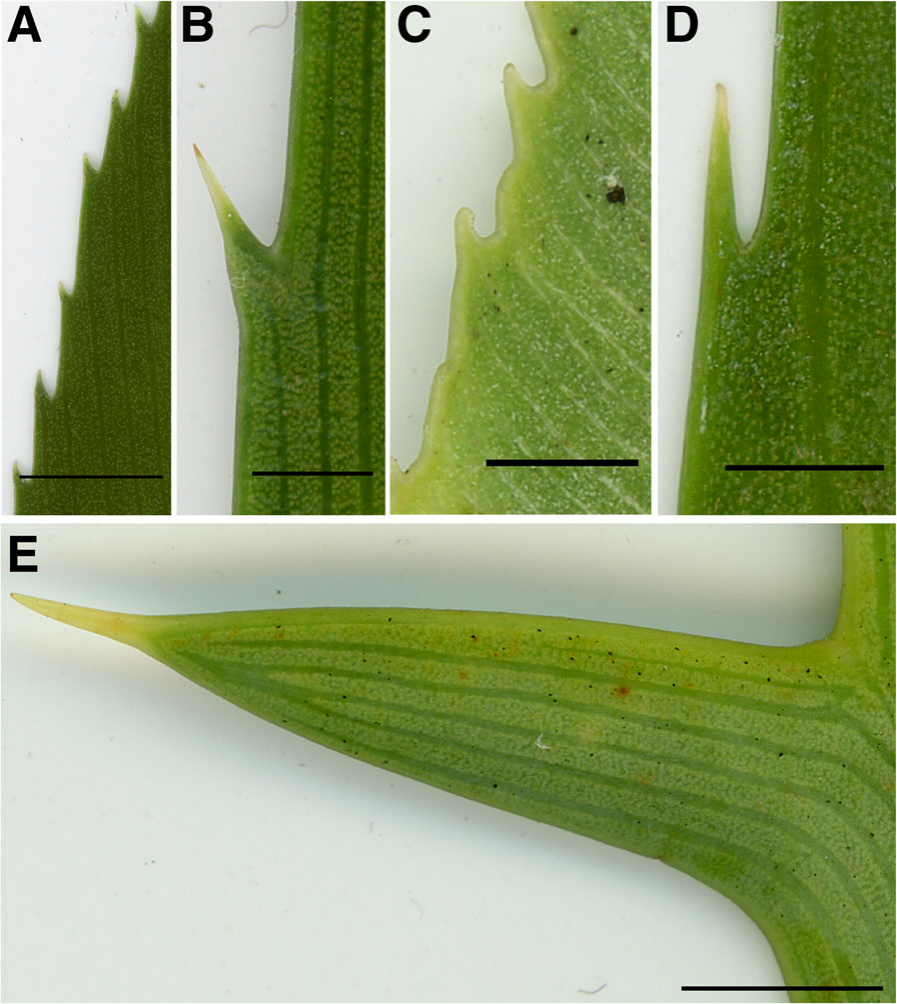
Details of teeth in different species of Zamiaceae, showing the “capped” appearance of the teeth and the difference between serrated margins, dentate margins and lobe-like teeth. **a** Leaf margin of *Bowenia serrulata*. **b** Leaf margin of *Encephalartos manikensis*. **c** Leaf margin of *Stangeria eriopus*. **d** Leaf margin of *Zamia neurophyllidia*. **d** Leaf margin of *Encephalartos horridus,* showing the lobe-like tooth. Scale bars: **a**, **e** 1 cm; **b**, **c**, **d** 0.25 cm

*Eobowenia* and *Bowenia* not only share significant and interesting characters in epidermal and cuticular anatomy, but also share commonalities at the macromorphological level, one being the serrate leaflet margin, which occurs in many extant cycads, such as some species of *Zamia* and *Stangeria* (Fig. 6). Marginal teeth are also present in a few species of *Encephalartos* (Fig. 6 b). In *Bowenia,* a serrate margin is present in both *B. serrulata* (Fig. 6 a) and individuals of *B. spectabilis* Hook. ex Hook.f. growing in more open environments [59], as well as in the fossil *B. eocenica* R.S.Hill [29] and other fossil members of the genus that have not yet been formally described [33]. The thickened, almost glandular-like aspect of the teeth in *Eobowenia* is compatible with the situation present in extant (Fig. 6) as well as fossil cycads (i.e. *Restrepophyllum*, [38]). In extant cycads, the thickened aspect of the tooth is given by a concentration of marginal fibres. The teeth in *Almargemia dentata,* on the other hand (Fig. 1) remind more closely of the lobe-like teeth present in some species of *Encephalartos* (Fig. 6 e). The basally converging veins in the leaflets are another character shared between *Eobowenia incrassata* and *Bowenia*. This character is also present in members of *Zamia,* but *Zamia* has articulated leaflets in contrast to the decurrent insertion of the leaflets in *Bowenia* and *Eobowenia*. The striations on the leaflets of *Eobowenia* remind of similar striations present in fossil representatives of *Bowenia* (described by [29] as “veinlets”), which correspond to interspersed fibres in the leaflets of extant *Bowenia.*

However, *Bowenia* and *Eobowenia* also differ in details that are mainly restricted to the morphology of their leaflets. All extant and extinct species of *Bowenia* are characterised by dichotomous venation, with veins ending at the margin. In the species with serrate leaflet margin, the veins commonly end in the teeth. In *Eobowenia*, the details of the venation are not clear from the material available, even if some dichotomies are potentially present on the specimen (Fig. 2 a, b).

Another striking difference between *Eobowenia* and *Bowenia* lies in the size of the leaflets. The two extant species of *Bowenia* have leaflets with length varying from 9 to 14 cm [59], which are markedly larger than the 0.6–1.0 cm long leaflets of *Eobowenia*. However, fossil leaves assigned to *Bowenia* commonly have rather short leaflets (e.g. *B. eocenica* and *B. papillosa* R.S.Hill: 3–4 cm; [33]). Extant *Bowenia* is characterised by bipinnate leaves, which are an autapomorphy of the genus, while the fragmentary nature of the leaflets of *E. incrassata* does not allow us to evaluate the character in this taxon.

Despite the striking similarities presented between *Eobowenia incrassata* and *Bowenia*, we refrain from assigning the specimens to *Bowenia*, mainly in the light of the differences outlined above, and considering the institution of the new genus *Eobowenia* to represent the best solution for the accommodation of this fossil taxon.

On the other hand, the differences and uncertainties in macromorphological characters do not preclude a relationship between the two genera. Regarding, for example, leaflet size, size variation is not uncommon among extant and fossil Cycadales. For example, in extant *Zamia*, leaflet length can vary from 1 to 8 cm in *Z. pygmaea* Sims [60] to 30–60 cm in *Z. wallisii* A.Braun. In the fossil genus *Ctenis*, leaflet length can vary from 1.5–3.5 cm in *C. nathorstii* Moeller [42] to 15–20 cm in *C. kaneharai* Yokoyama [55].

### *Phylogenetic evidence for the placement of* Eobowenia *and* Almargemia

Our investigation is not the first to hypothesise a link between *Eobowenia (Almargemia) incrassata* and *Bowenia.* In their phylogenetic analysis of extant and fossil cycads, Martinez et al. [48] retrieved a maximum parsimony tree with *Almargemia* (predominantly coded after *A. incrassata*) as sister to *Bowenia* plus *Stangeria* and *Mesodescolea*. However, such relationship does not receive any support from the bootstrap analysis, and it is not retrieved in other analyses of morphology, which consider *Almargemia* predominantly coded for *A. incrassata* [61, 62].

Using the topology from Salas-Leiva et al. [7] and the modified matrix from Martinez et al. [48] as a backbone, the best placement for *Eobowenia* is as sister to *Bowenia* (Fig. 5 a). However, alternative placements are possible at the cost of only one or two steps more. This could be due to the low number of characters coded for *Eobowenia* (21 characters out of 89), and the few informative epidermal characters linking the different clades of the Zamiaceae. If we consider the placements which are only one step longer, these placements imply that the unique characters of the stomatal complex of *Eobowenia* (guard cells at level with epidermis and monocyclic stomatal complexes) either evolved independently in this taxon and in *Bowenia* (if *Eobowenia* is placed as sister to the Ceratozaminae or the Encephalartinae), or represent a potentially plesiomorphic status of all Zamiaceae except *Dioon.* This would imply that all the similarities of the stomatal complexes of the Encephalartinae and *Dioon* could represent parallel evolution of sunken, protected guard cells.

Our phylogenetic analyses based on the Martinez et al. [48] matrix retrieve a relationship between *Eobowenia* and *Bowenia* in both the MP and BI analyses of the morphological data, with *Eobowenia* being sister to the Stangeriaceae sensu Stevenson [6] but such relationships only receive low support in the BI analysis. In the MP analysis this is partially due to the uncertainties surrounding the relationships between *Bowenia* and *Stangeria* and many other fossil taxa with peculiar character combinations, such as *Kurtziana, Mesosingeria, Sueria* and *Pseudoctenis.* When information from the molecular analysis of Salas-Leiva et al. [7] is added, resulting in the breakup of the Stangeriaceae, *Eobowenia* is preferentially retrieved as sister to *Bowenia* instead of *Stangeria.* The characters linking *Eobowenia* and *Bowenia* in these topologies regard the unique structure of the stomatal apparatus, which combines the flush guard cells with the lack of encircling cells. The combined analysis using a Bayesian framework retrieves the strongest support for the sister relationship of the two genera. This is in our knowledge the first attempt of integrating morphology and molecular data in a matrix that includes fossil taxa in the Cycadales, and shows the potential of this practice to resolve some of the uncertainties in the relationships between extant and fossil cycads.

The placement of *Almargemia*, on the other hand, is much more uncertain, with no clear placement in any of the analyses. However, a sister relationship between *Eobowenia* and *Almargemia* is never retrieved.

Our phylogenetic analyses show that the link between *Eobowenia* and *Bowenia* is the best hypothesis to explain the relationship between the fossil taxon and the diversity of the Cycadales, even when adopting a conservative approach to its macromorphological character coding. Such phylogenetic evidence, which is lacking for many fossil cycads that have been linked with extant groups, such as *Restrepophyllum* [38] and *Austrozamia* [25], as well as for the many fossil leaves assigned to extant genera [21–24, 26–28], make *Eobowenia* a reliably placed cycad fossil foliage.

Such a placement is also compatible with at least some of the inferred age for the divergence of *Bowenia* based on molecular dating. The age of the deposition of the Anfiteatro de Ticó Formation, where *Eobowenia* is found, is very well constrained to 118.23 ± 0.09 Ma [63] or 116.85 ± 0.26 Ma [64] representing an Aptian (Lower Cretaceous) age, which is compatible with the ages inferred for the stem of *Bowenia* by Nagalingum et al. [17] using a relaxed log-normal clock and by Condamine et al. [16] using the favoured birth-death prior with both the calibration implemented, but is older than the dates retrieved by Salas-Leiva et al. [7] (Table 2). This probable early divergence of the genus *Bowenia* is, however, compatible with the phylogenetic placement retrieved by the multilocus analysis of Salas-Leiva et al. [7], which sees *Bowenia* as sister to all the other Zamiaceae apart from *Dioon.* A Cretaceous stem history of *Bowenia/Eobowenia* is also compatible with the presence of cuticle indistinguishable from modern *Bowenia* in the Upper Cretaceous of Central Australia [32].

**Table 2:** Summary of the dates of divergence between *Bowenia* and its sister group in the more recent phylogenetic analyses that included a molecular dating analyses

	Analysis	Median age	95% HPD
Nagalingum et al. [17]		102	64.6–137.2
Salas-Leiva et al. [7]		74.8	56.4–91.0
Condamine et al. [16]	Traditional fossil set	116.3	76.7–160.8
Condamine et al. [16]	New fossil set	156.1	107–207.9

### Eobowenia *and the biogeography of* Bowenia

The occurrence of a potential sister of *Bowenia* in the Early Cretaceous of Patagonia helps to strengthen some of the hypotheses around the biogeography of *Bowenia*. Until now, the phylogenetic isolation of *Bowenia*, as well as the presence of fossil records limited to Australia, had complicated the resolution of the biogeography of the genus. Indeed, Salas-Leiva et al. [7] retrieved two different results in their analysis: using S-DIVA, they retrieved an ancestral area including Australia, Africa and Mexico for the stem of *Bowenia*, while their DEC analysis hypothesises a model of stasis in Australia. The presence of *Eobowenia* in Patagonia during a period of connectivity between southern America and Australasia supports the hypothesis of a Gondwanan distribution for the stem of the group, with subsequent extinction shaping the current Australian endemic distribution. *Bowenia* would indeed represent yet another case of eastern survival [65]. Even if we know that some cycads persisted in southern South America until the Palaeocene [25], the identification of the precise timing of the extinction of *Eobowenia* in South America is hindered by the potential rarity of this fossil leaf type in the record. However, it is clear that this fossil represents another important clue to the biogeography of Gondwana coming from Patagonia [20].

## Conclusions

Based on our reinvestigation, we conclude that the leaves assigned by Archangelsky [43] to *Almargemia incrassata* are best accommodated in the new genus *Eobowenia.* A phylogenetic analysis indicated that *Eobowenia* could represent the sister group of extant *Bowenia*. This placement bears interesting implications for the biogeography of *Bowenia,* which could represent another example of an Australian relict of a previously widespread Gondwanan taxon.

## Additional files

Additional file 1: Table S1. List of the species with accession numbers and provenance used as comparative material in this study. MBC: Montgomery Botanical Center; NAP: Orto Botanico di Napoli; Z: Herbarium Zurich. (DOCX 14 kb)

Additional file 2: Figure S1. Maximum parsimony reconstruction of character evolution on the combined molecular-morphological Bayesian tree for character 50 (A), 53 (B) and 89 (C). (PDF 97 kb)

Additional file 3: Figure S2. Isolated cuticles of *Cycas rumphii* (A, B) and *Dioon merolae* (C, D) stained with Auramine O. Scale bar: 100 μm. (PNG 2853 kb)

Additional file 4: Figure S3. Isolated cuticles of *Ceratozamia mexicana* (A, B), and *Stangeria eriopus* (C, D), *Microcycas calocoma* (E, F) and *Zamia portoricensis* (G, H) stained with Auramine O. Scale bar: 100 μm. (PNG 5239 kb)

Additional file 5: Figure S4. Isolated cuticles of *Macrozamia plurinervia* (A, B), *Lepidozamia hopei* (C, D), and *Encephalaros manikensis* (E, F) stained with Auramine O. Scale bar: 100 μm. (PNG 3705 kb)

Additional file 6: Figure S5. Stomatal complexes of *Stangeria eriopus* (A), and *Bowenia spectabilis* (B). Encircling cells (ec) and subsidiary cells (sc) are underlined. Scale bar: 100 μm. (PNG 1008 kb)

Additional file 7: Figure S6. Epifluorescence micrograph of partially digested epidermis of *Encephalartos ferox* (A) and *Macrozamia plurinervia* (B), showing the presence of a thickened substomatal apparatus. (TIFF 3701 kb)

Additional file 8: Zip file containing matrices and trees. (ZIP 1603 kb)

## Abbreviations

BI: Bayesian Inference
MP: Maximum parsimony
NHM: Natural History Museum, London
NRM: Swedish Museum of Natural History
