# Supplementary material for: Eobowenia gen. nov. from the Early Cretaceous of Patagonia: indication for an early divergence of Bowenia?
Source: BMC Evol Biol. 2017 Apr 7;17:97. doi: 10.1186/s12862-017-0943-x (PMC5383990; doi:10.1186/s12862-017-0943-x)
Supplement: Supplementary file 1 — List of the species with accession numbers and provenance used as comparative material in this study. MBC: Montgomery Botanical Center; NAP: Orto Botanico di Napoli; Z: Herbarium Zurich. (DOCX 14 kb) [file 12862_2017_943_MOESM1_ESM.docx]

| Species | ID | Origin |
| --- | --- | --- |
| *Bowenia serrulata* (W.Bull) Chamb. | 94632*G | MBG |
| *Bowenia serrulata* (W.Bull) Chamb. | 2004865*P | MBG |
| *Bowenia spectabilis* HOok. ex Hook.f. | 9367*A | MBG |
| *Ceratozamia mexicana* Brongn. | CM20 | NAP |
| *Cycas rumphii* Miq. | 415-121 | NAP |
| *Dioon edule* Lindl. | BL_NAP_01 | NAP |
| *Dioon merolae* De Luca, Sabato & Vázq.Torres | BL_NAP_02 | NAP |
| *Encephalartos horridus* (Jacq.) Lehm. | 9838*A | MBG |
| *Encephalartos laurentianus De Wild.* | 383-0-1 | NAP |
| *Zamia portoricensis* (Gilliland) Gilliland | 385-0-1 | NAP |
| *Encephalartos manikensis* (Gilliland) Gilliland | 2006198*A | MBG |
| *Encephalartos transvenosus* Stapf & Burtt Davy | ET01 | NAP |
| *Lepidozamia hopei* (W.Hill) Regel | Z000102580 | Z |
| *Macrozamia plurinervia* (L.A.S.Johnson) D.L.Jones | 437-8-1 | NAP |
| *Microcycas calocoma* (Miq.) A.DC*.* | 20010812*D | MBG |
| *Stangeria eriopus* (Kunze) Baill. | Z000102581 | Z |
| *Stangeria eriopus* (Kunze) Baill. | 80727*E | MBG |
| *Zamia neurophyllidia* D.W.Stev*.* | 86567*GGG | MBG |
| *Zamia portoricensis* Urb. | 528-0-1 | NAP |

**Table S1**: List of the species with accession numbers and provenance used as comparative material in this study. MBC: Montgomery Botanical Center; NAP: Orto Botanico di Napoli; Z: Herbarium Zurich.
