# Supplementary figures and images for: Eobowenia gen. nov. from the Early Cretaceous of Patagonia: indication for an early divergence of Bowenia?
Source: BMC Evol Biol. 2017 Apr 7;17:97. doi: 10.1186/s12862-017-0943-x (PMC5383990; doi:10.1186/s12862-017-0943-x)

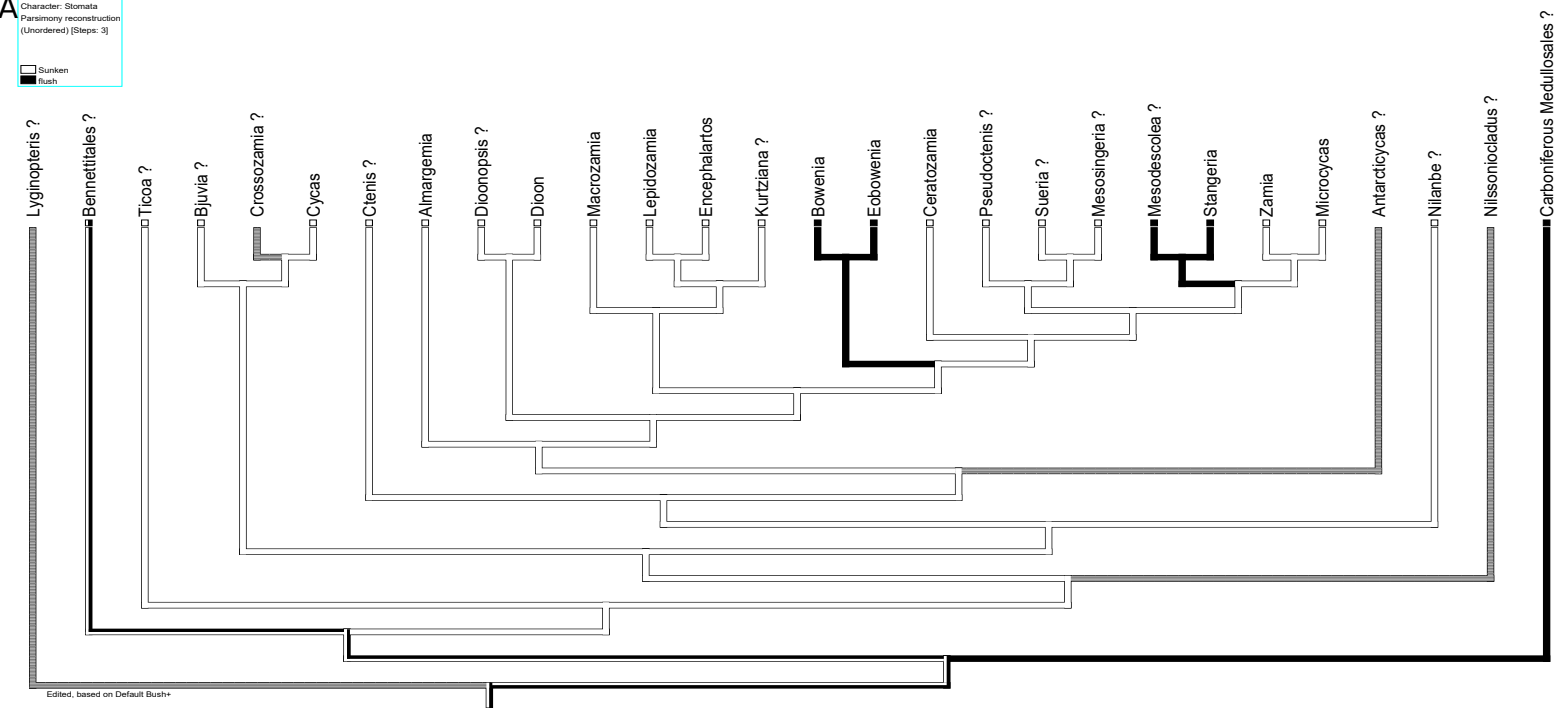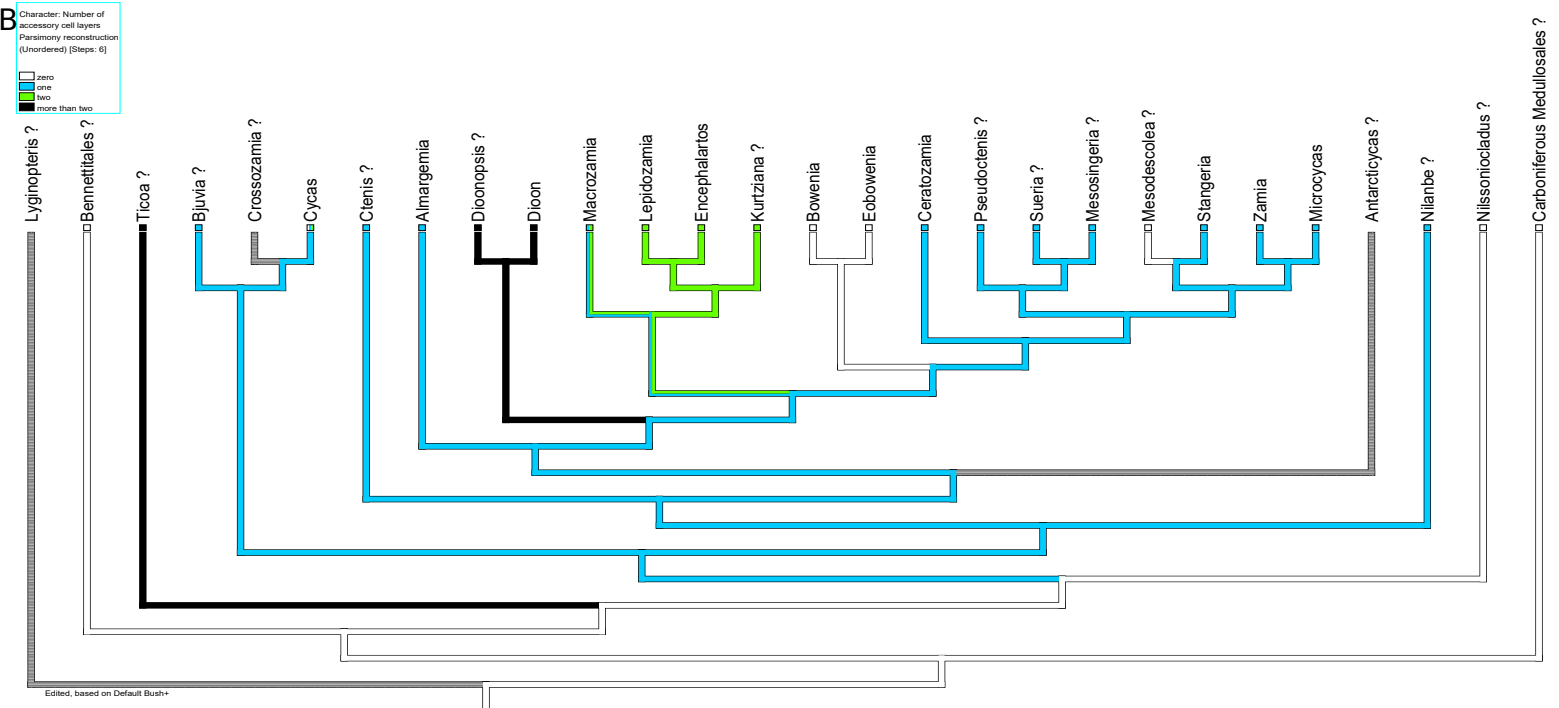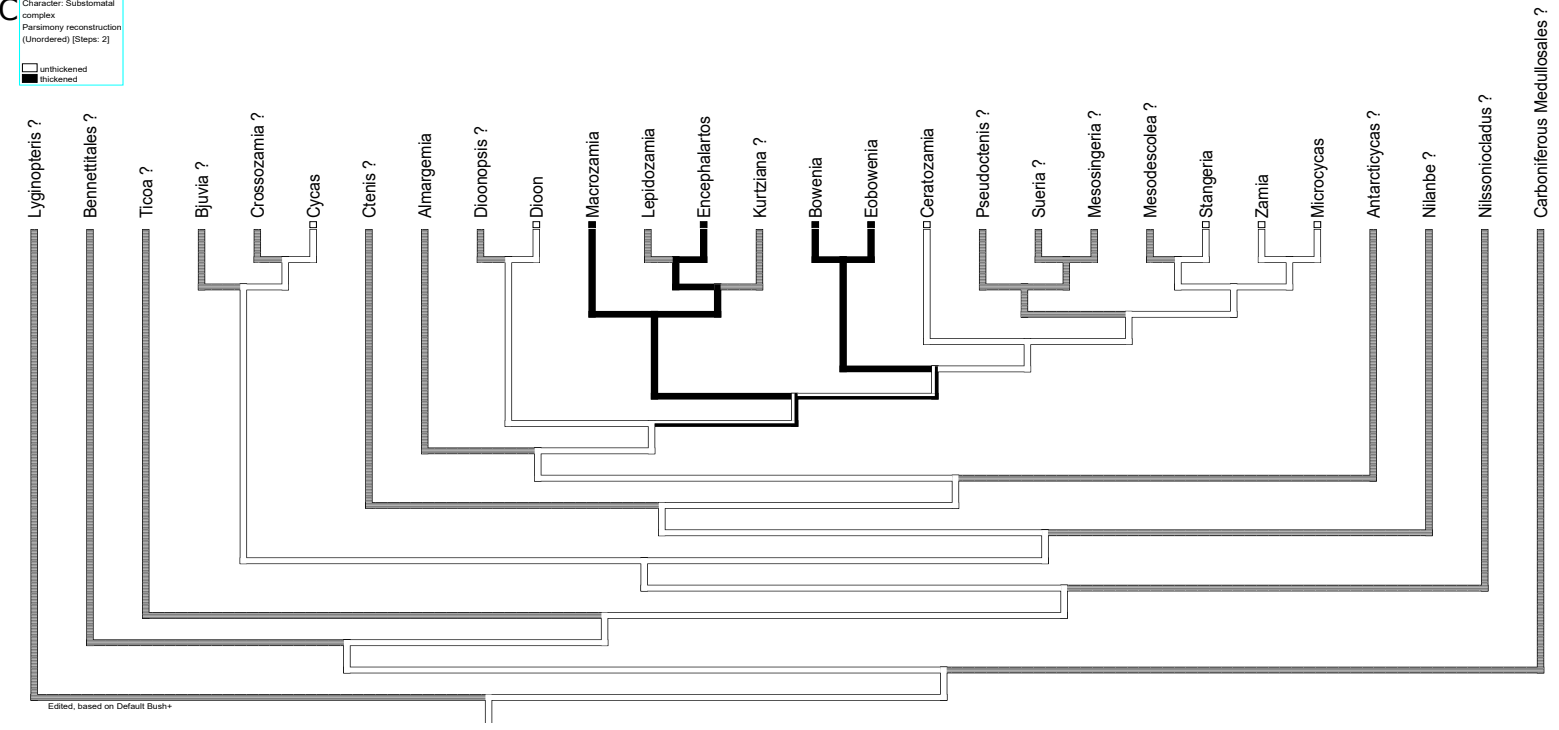

Supplement: Supplementary file 2 — Maximum parsimony reconstruction of character evolution on the combined molecular-morphological Bayesian tree for character 50 (A), 53 (B) and 89 (C). (PDF 97 kb) [file 12862_2017_943_MOESM2_ESM.pdf]

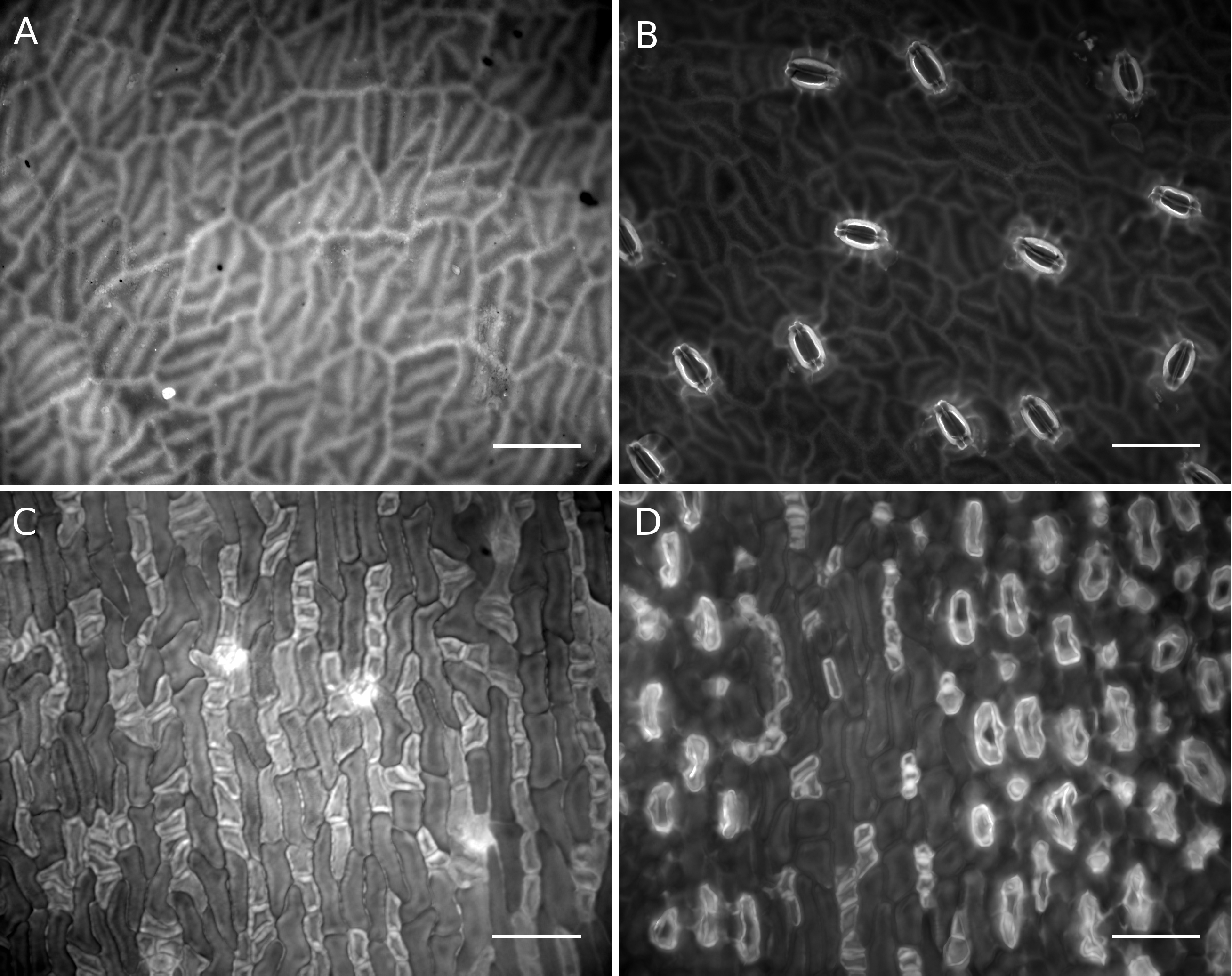

Supplement: Supplementary file 3 — Isolated cuticles of Cycas rumphii (A, B) and Dioon merolae (C, D) stained with Auramine O. Scale bar: 100 μm. (PNG 2853 kb) [file 12862_2017_943_MOESM3_ESM.png]

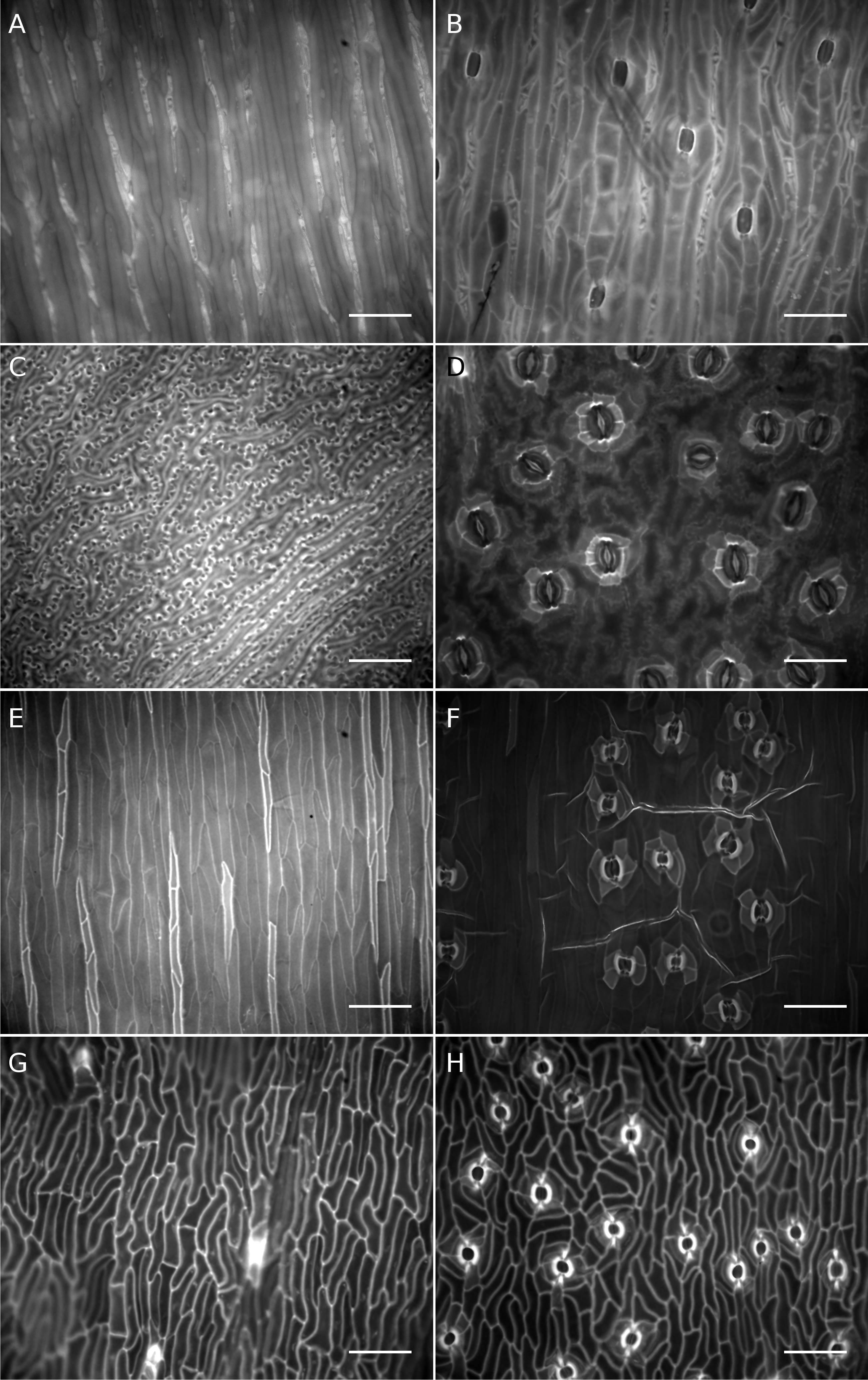

Supplement: Supplementary file 4 — Isolated cuticles of Ceratozamia mexicana (A, B), and Stangeria eriopus (C, D), Microcycas calocoma (E, F) and Zamia portoricensis (G, H) stained with Auramine O. Scale bar: 100 μm. (PNG 5239 kb) [file 12862_2017_943_MOESM4_ESM.png]

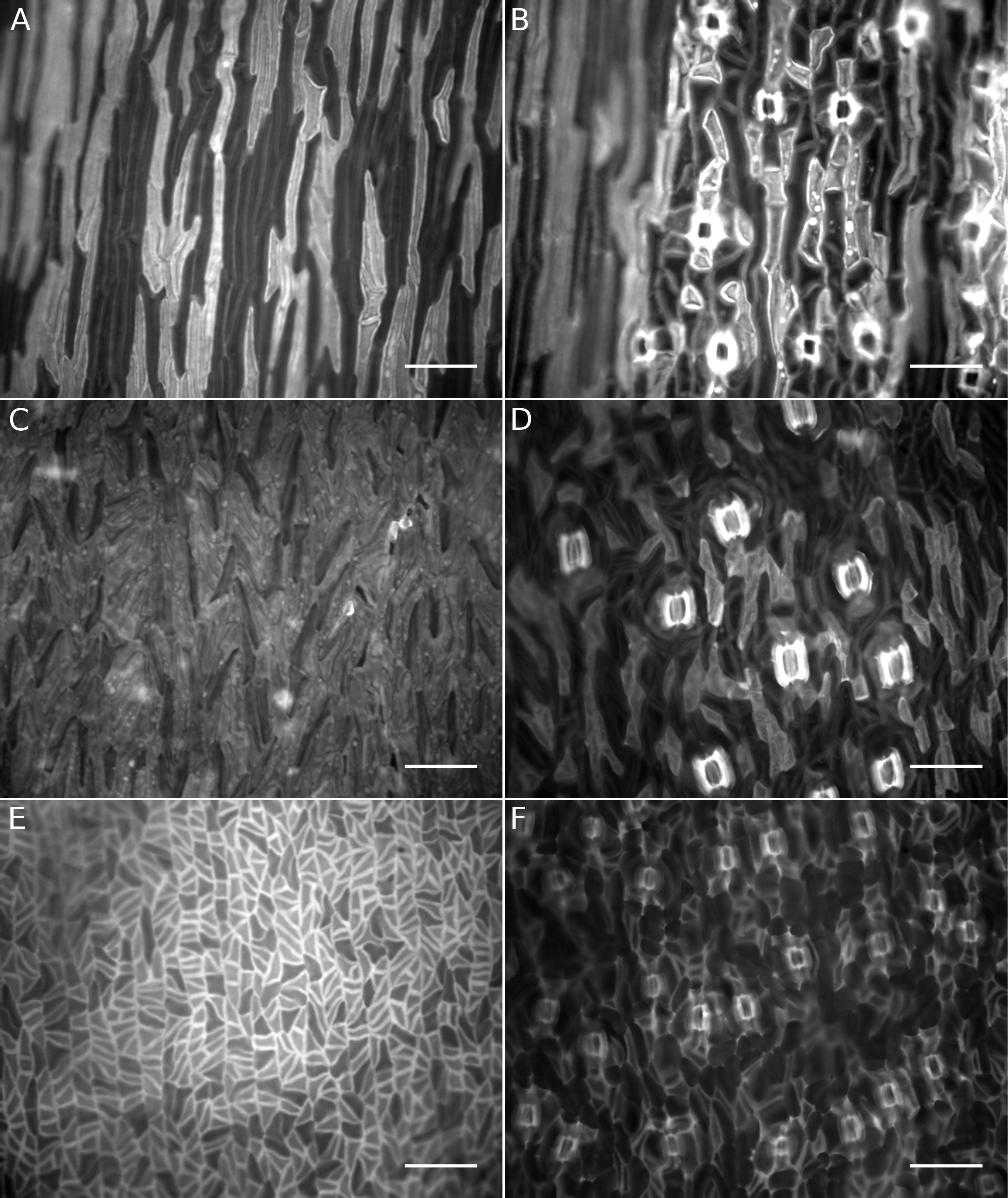

Supplement: Supplementary file 5 — Isolated cuticles of Macrozamia plurinervia (A, B), Lepidozamia hopei (C, D), and Encephalaros manikensis (E, F) stained with Auramine O. Scale bar: 100 μm. (PNG 3705 kb) [file 12862_2017_943_MOESM5_ESM.png]

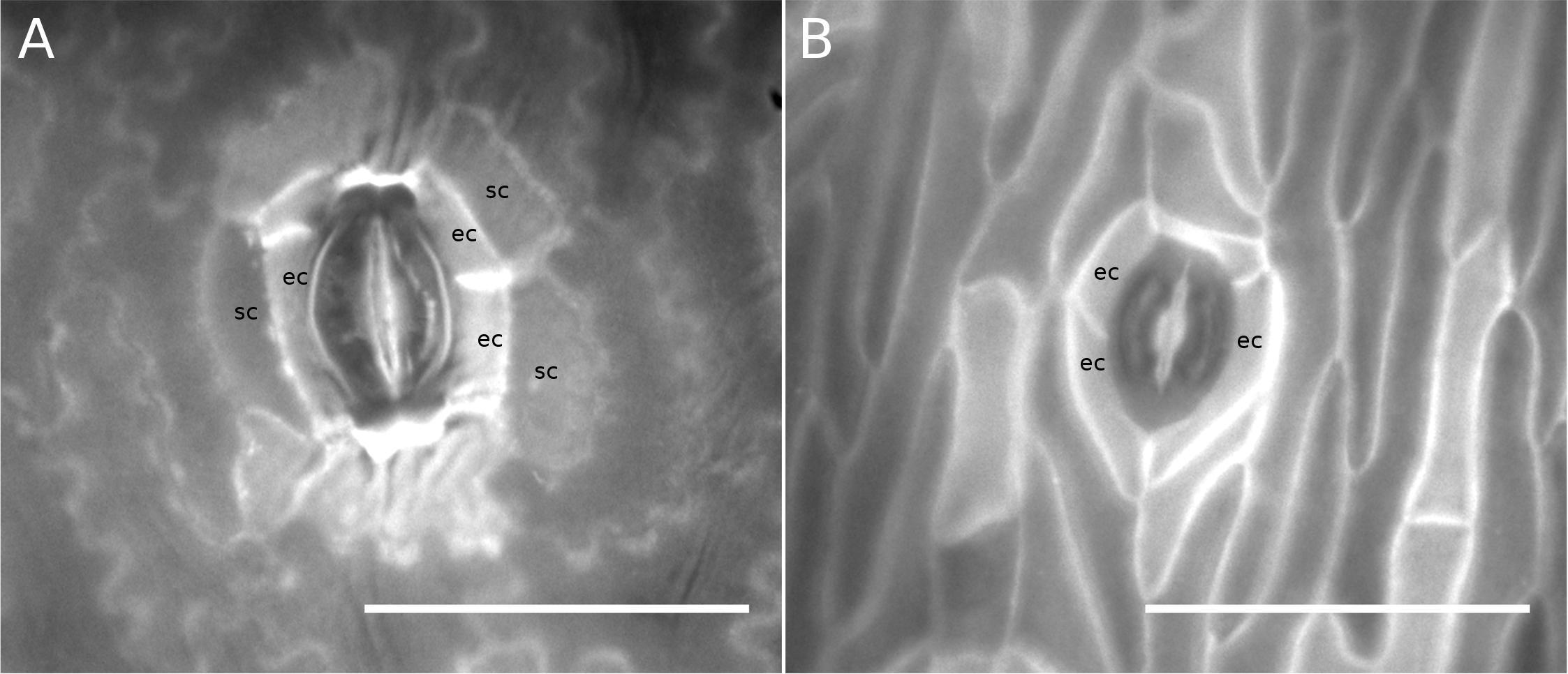

Supplement: Supplementary file 6 — Stomatal complexes of Stangeria eriopus (A), and Bowenia spectabilis (B). Encircling cells (ec) and subsidiary cells (sc) are underlined. Scale bar: 100 μm. (PNG 1008 kb) [file 12862_2017_943_MOESM6_ESM.png]

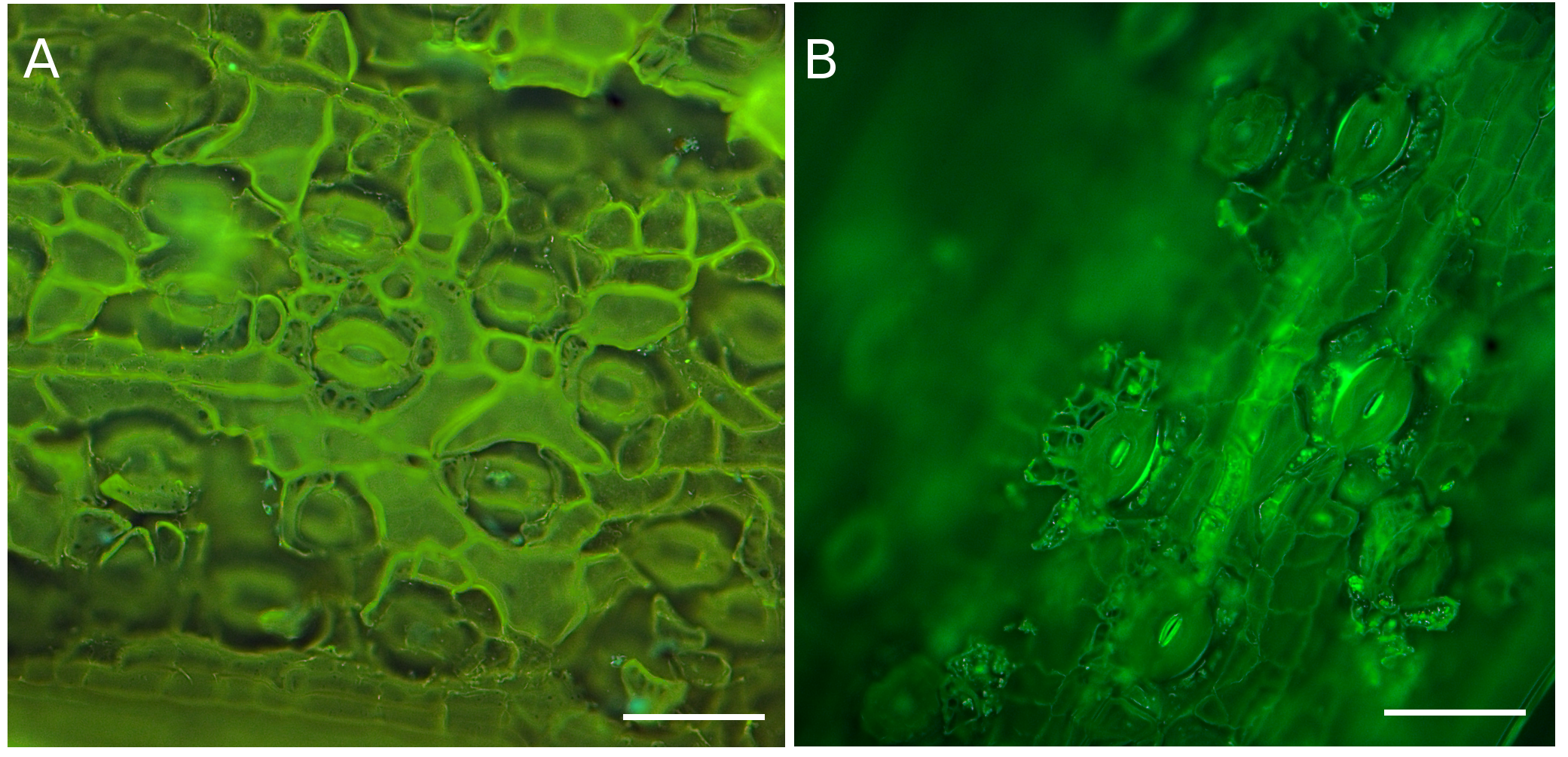

Supplement: Supplementary file 7 — Epifluorescence micrograph of partially digested epidermis of Encephalartos ferox (A) and Macrozamia plurinervia (B), showing the presence of a thickened substomatal apparatus. (TIFF 3701 kb) [file 12862_2017_943_MOESM7_ESM.tiff]
